# O avanço da radioterapia no tratamento das lesões metastáticas da coluna

**DOI:** 10.1055/s-0045-1810031

**Published:** 2025-08-18

**Authors:** Brian Guilherme Monteiro Marta Coimbra, Alice Roxo Nobre de Souza e Silva, Cauã Bhering Soares, Caio César Nogueira de Figueiredo, Manuel Salvado Macamo, Alexandre Fogaça Cristante

**Affiliations:** 1 Grupo de Coluna, Instituto do Câncer do Estado de São Paulo, Hospital das Clínicas da Faculdade de Medicina da Universidade de São Paulo, São Paulo, SP, Brasil; 2 Grupo de Radioterapia, Instituto do Câncer do Estado de São Paulo, Hospital das Clínicas da Faculdade de Medicina da Universidade de São Paulo, São Paulo, SP, Brasil; 3 Complementação Especializada do Grupo de Coluna, Hospital das Clínicas da Faculdade de Medicina da Universidade de São Paulo, São Paulo, SP, Brasil; 4 Departamento de Ortopedia e Traumatologia, Faculdade de Medicina da Universidade de São Paulo, São Paulo, SP, Brasil

**Keywords:** coluna vertebral/cirurgia, metástase neoplásica, radioterapia, neoplasm metastasis, radiotherapy, spine/surgery

## Abstract

A doença metastática da coluna vertebral tem se tornado cada vez mais comum por conta do avanço das terapias sistêmicas oncológicas e do aumento na sobrevida geral dos pacientes com câncer. O tratamento dessa enfermidade é paliativo, mas a própria acepção deste termo evoluiu e hoje consiste em um controle melhor e mais efetivo da doença epor períodos mais longos. Enquanto no passado as técnicas cirúrgicas e radioterápicas, principalmente a radioterapia convencional por feixe externo (cEBRT, do inglês *conventional external beam radiation therapy* ), eram ineficazes ou mórbidas para o controle de muitos tipos de tumores, hoje, com o surgimento e avanço da radiocirurgia estereotáxica corporal (SBRT, do inglês *stereotactic body radiation therapy* ), esse cenário mudou drasticamente. A cirurgia para o manejo da doença metastática ainda tem lugar em estratégias associadas com a radioterapia, mas sofreu grande atualização de propósito e técnica. Todavia, apesar de todo o avanço técnico, a radioterapia apresenta toxicidade e efeitos colaterais que precisam ser considerados. Neste artigo, fornecemos revisão atualizada que versa desde o histórico até as inovações no tratamento das metástases vertebrais, com foco em seus benefícios, indicações e impacto clínico.

## Abstract (en)

Metastatic spinal disease has become increasingly common due to advances in systemic oncological therapies and the increase in the overall survival of cancer patients. Its treatment is palliative, but the meaning of this term has evolved and, today, consists of better and more effective condition control over longer periods. In the past, surgical and radiotherapy techniques, especially conventional external beam radiation therapy (cEBRT), were ineffective or morbid for controlling many types of tumors. Now, the emergence and advancement of stereotactic body radiation therapy (SBRT) drastically changed this scenario. Surgery for managing metastatic disease remains significant in radiotherapy-associated strategies, but its purpose and technique have undergone major updates. However, despite all technical advances, radiotherapy has toxicity and side effects that warrant consideration. In this article, we provide an updated review covering everything from history to innovations in treating spinal metastases, focusing on their benefits, indications, and clinical impact.

## Introdução

Nas últimas décadas, com o avanço científico no campo oncológico e a maior disponibilidade de terapias sistêmicas mais efetivas, a sobrevida dos pacientes com câncer, em geral, tem aumentado, e com ela também a prevalência de doença no contexto metastático. 1 A coluna vertebral é o local mais comum de metástases no esqueleto devido, entre outros fatores, à sua rica vascularização e à abundância de tecido medular ósseo. Essas lesões podem causar dor, instabilidade mecânica e, em casos mais graves, compressão medular (ou da cauda equina), representando um desafio terapêutico interdisciplinar. O tratamento das metástases é paliativo, com objetivos diversificados como controle álgico, recuperação neurológica, melhora de qualidade de vida, controle do volume tumoral e viabilização de terapia sistêmica.

A radioterapia é uma das modalidades fundamentais para o manejo das metástases ósseas na coluna e o desenvolvimento de tecnologias avançadas revolucionou o cenário terapêutico, permitindo abordagens mais eficazes, seguras e individualizadas. A radioterapia convencional por feixe externo (cEBRT, do inglês *conventional external beam radiation therapy* ) é a abordagem padrão, mas nos últimos anos a radioterapia estereotáxica corporal corporal (SBRT, do inglês *stereotactic body radiation therapy* ) emergiu como uma alternativa eficaz, particularmente para controle local e em tumores radiorresistentes. 1

Este artigo apresenta uma revisão atualizada que versa desde o histórico até as inovações no tratamento das metástases vertebrais, com foco em seus benefícios, indicações e impacto clínico.

## Breve histórico da evolução do tratamento das metástases da coluna vertebral

Nos últimos 50 anos, o tratamento paliativo das metástases evoluiu no sentido de obter efeitos mais duráveis e com menos morbidade. Nos anos 1970, as técnicas cirúrgicas ainda careciam de maior refinamento e as descompressões resultavam em instabilidades grosseiras. Nesse contexto, a cEBRT ganhou campo como estratégia de tratamento, apesar de baixa eficácia em casos de tumores sólidos. Havia a canalização de radiação de modo fracionado com preocupação de poupar órgãos nobres como a medula, aparelho digestivo e rins, mas com resultados insatisfatórios no controle de doença. Em 1978, Gilbert et al **.** 2 demonstraram a radiossensibilidade e a radiorresistência dos tumores baseadas em sua histologia, sendo os tumores hormônio-dependentes e os hematológicos os mais responsivos à radiação.

Com o avanço das técnicas de instrumentação nos anos de 1990, a cirurgia retomou papel importante na abordagem dos pacientes com tumores metastáticos na coluna vertebral, inaugurando uma era de grandes ressecções e reconstruções vertebrais. Nos anos 2000, a transição de materiais como aço inoxidável para o titânio possibilitou melhores avaliações por exames de imagem e, portanto, melhores planejamento e execução do tratamento radioterápico complementar nos casos em que a cirurgia se fazia imperativa.

Em 2005, Patchell et al. 3 publicaram um ensaio prospectivo randomizado comparando a cEBRT à cirurgia seguida de cEBRT em pacientes com compressão de alto grau do canal vertebral causada por tumores sólidos radiorresistentes. O braço do estudo em que a cirurgia seguida de radioterapia foi aplicada apresentou resultados muito superiores, de modo que, com este e vários outros estudos que foram então conduzidos, o Spine Oncology Study Group (SOSG) recomendou esta abordagem combinada como a estratégia mais efetiva a ser adotada nestes casos. 3

Nos últimos 15 anos, o desenvolvimento da SBRT revolucionou o paradigma de manejo das lesões secundárias na coluna. Por definição, ela consiste na administração de radiação em alta dose, pouco fracionada, que varia de 16 a 24 Gy em fração única ou 24 a 40 Gy em 2 a 5 frações. Essa radiação é aplicada com técnicas conformacionais, isto é, com demarcação do sítio alvo de modo mais preciso, com auxílio de imagens, poupando tecidos saudáveis adjacentes. Esta estratégia supera a radiorresistência histológica dos tumores, levando a um controle biológico local efetivo.

O advento descrito acima limitou o papel das grandes ressecções cirúrgicas de outrora. Os tumores metastáticos que não comprimem a medula, radiorresistentes, em doença oligometastática, que antes eram tratados com ressecções *en bloc* , hoje recebem, como primeira linha de tratamento, a radiocirurgia. Enquanto as demais indicações cirúrgicas se mantiveram as mesmas desde o estudo de Patchell, o avanço da radioterapia resultou na mudança do objetivo da cirurgia. Se no passado havia uma preocupação com o volume ressecado, hoje se destaca a cirurgia de separação, na qual se cria um espaço de 2 a 3 mm entre as estruturas neurais e a massa tumoral, que então pode ser irradiada com segurança e eficácia.

Os grandes contrapontos da radioterapia são toxicidade e efeitos colaterais. Danos a tecidos saudáveis e nobres, neurites, mielites e fraturas vertebrais compressivas são efeitos que, apesar de cada vez menos frequentes com o progressivo refinamento tecnológico, ainda existem.

## Fisiopatologia e mecanismo de Ação

As metástases ósseas resultam de um processo biológico altamente complexo, iniciado pela disseminação hematogênica de células tumorais que, ao migrarem do sítio primário, invadem tecidos adjacentes, entram na circulação e se alojam preferencialmente na medula óssea. Este microambiente ósseo, rico em fatores de crescimento, como *transforming growth factor-beta* (TGF-β) e *platelet-derived growth factor* (PDGF), promove a proliferação das células tumorais. Essas células secretam proteínas, como a *parathyroid hormone-related peptide* (PTHrP), que ativam osteoclastos por meio da sinalização do receptor ativador do fator nuclear kappa B (RANK, do inglês *receptor activator of nuclear factor-kappa B* ,)/ligante RANK (RANKL, do inglês *RANK ligand* ), intensificando a reabsorção óssea. A degradação do osso, por sua vez, libera mais fatores de crescimento armazenados na matriz, perpetuando um ciclo vicioso que alimenta a progressão tumoral, intensifica a destruição tecidual e amplifica o estímulo nociceptivo.

Do ponto de vista fisiopatológico, a dor nas metástases ósseas é mediada por múltiplos mecanismos. Dentre eles, destacam-se a compressão de fibras nervosas periféricas, a liberação de mediadores inflamatórios, como interleucina-1 (IL-1), fator de necrose tumoral-alfa (TNF-α, do inglês *tumor necrosis factor-alpha* ) e prostaglandinas, e o aumento da pressão intraóssea devido à infiltração tumoral. Essas alterações são exacerbadas por microfraturas e deformidades ósseas, que amplificam os estímulos nociceptivos e promovem dor persistente e de difícil controle clínico. 4

A radioterapia exerce efeitos terapêuticos multifacetados no tratamento das metástases ósseas, incluindo a indução de apoptose das células tumorais, resultando na redução da carga tumoral e no alívio da pressão intraóssea. Além disso, há uma supressão significativa da atividade osteoclástica, promovendo estabilização da reabsorção óssea e facilitando o processo de reparo tecidual. A radioterapia também modula o microambiente inflamatório ao reduzir a presença de células inflamatórias e citocinas pró-inflamatórias, o que explica o rápido alívio da dor observado em muitos casos, frequentemente nas primeiras 24 horas após o início do tratamento.

Outro efeito importante é a estimulação da ossificação em lesões osteolíticas, que contribui para a restauração parcial da integridade estrutural óssea. Além disso, há evidências de que a radioterapia pode modular diretamente a excitabilidade de fibras nervosas periféricas na região irradiada, o que atenua a dor neuropática associada às metástases ósseas.

## Radioterapia convencional paliativa

Tradicionalmente, a cEBRT utiliza doses como 8 Gy em dose única ou esquemas fracionados (20 Gy em 5 frações ou 30 Gy em 10 frações). Em 2011, a American Society for Therapeutic Radiology and Oncology (ASTRO) publicou uma diretriz baseada em evidências para o tratamento paliativo de metástases ósseas. A análise de diversos ensaios clínicos randomizados prospectivos demonstrou equivalência no alívio da dor para estes diferentes esquemas de dose frequentemente utilizados em pacientes com lesões ósseas dolorosas sem história prévia de radioterapia, com toxicidades tardias aceitáveis e similares. A diferença observada foi com relação à taxa de retratamento por recorrência da dor, sendo significativamente maior no tratamento com fração única (8% *versus* 20%). 5 Na versão atualizada da diretriz, além de reforçar as questões anteriores, estabeleceu-se a recomendação de que o intervalo de um mês seria o prazo mínimo seguro para reirradiação em uma mesma região ou lesão. 6

Uma revisão sistemática e uma metanálise publicados posteriormente reforçaram as evidências quanto à equivalência da radioterapia de fração única (SFRT, do inglês *single-fraction radiotherapy* ) em comparação à radioterapia de múltiplas frações (MFRT, do inglês *multiple-fraction radiotherapy* ) em termos de eficácia no controle da dor, fratura patológica e compressão medular. As taxas de retratamento demonstram melhores resultados com MFRT ao longo do tempo de seguimento. 7 8 A avaliação desses estudos traz taxas gerais de resposta à dor de cerca de 60% e de resposta completa de 10 a 25% com o uso da cEBRT paliativa, com durabilidade de resposta de cerca de 4 meses. 9

## Radioterapia estereotáxica corpórea paliativa

A SBRT representa um marco no tratamento das metástases vertebrais. Essa técnica permite a administração de doses ablativas com alta precisão, minimizando a exposição de estruturas críticas próximas às lesões, como a medula espinhal. Além disso, o escalonamento de dose proporciona melhores taxas de controle da dor quando comparado à cEBRT. Em uma diretriz publicada este ano pela European Society of Therapeutic Radiology and Oncology (ESTRO), após revisão sistemática de estudos prospectivos e retrospectivos, a resposta geral e completa à dor após SBRT da coluna foi de 83,2% e 43,5%, respectivamente. 10

Um estudo randomizado de fase II comparou a durabilidade analgésica da SBRT (12 a 16 Gy em dose única) à radioterapia paliativa convencional (30 Gy em 10 frações) em pacientes com metástases ósseas dolorosas predominantemente não vertebrais. O estudo demonstrou que a SBRT não era inferior ao tratamento convencional em taxa de resposta geral à dor, além de apresentar resultados semelhantes de toxicidade aguda e taxas de fratura. 11 Essas descobertas representam a primeira evidência prospectiva randomizada a sugerir que a SBRT poderia se tornar o padrão de tratamento para pacientes, com boa eficácia, maior expectativa de vida e metástases ósseas limitadas.

Na investigação dos desfechos da radioterapia ablativa exclusivamente em metástases sintomáticas na coluna, Sahgal et al. 11 conduziram um estudo de fase 2/3 em centros do Canadá e da Austrália. Foram 229 pacientes randomizados entre SBRT, na dose de 24 Gy em 2 frações, e cEBRT, com 20 Gy em 5 frações. As lesões deveriam ser confirmadas por ressonância magnética, não comprometer mais do que três segmentos vertebrais contíguos, sem nenhuma compressão medular ou de cauda equina e um escore de instabilidade (Spine Instability Neoplastic Score, SINS) menor que 12. Os pacientes incluídos apresentavam índice de 0 a 2 pelo Eastern Cooperative Oncology Group (ECOG) e um quadro de dor definida como 2 pontos ou mais no Brief Pain Inventory. Com um desfecho primário de resposta completa para dor em 3 meses após a radioterapia, o estudo mostrou o benefício significativo da SBRT, com 35% dos pacientes deste grupo apresentando resposta, em comparação a 14% do grupo da cEBRT. Não houve diferença significativa em eventos adversos, incluindo o risco de fratura vertebral, e o acompanhamento de 6 meses mostrou resposta duradoura. 12

Mais recentemente, o estudo de fase 3 NRG-Oncology/RTOG 0631 comparou as duas técnicas de radioterapia, randomizando 339 pacientes com até 3 locais de metástases vertebrais para receber o tratamento em uma única fração, com SBRT (16 ou 18 Gy, no segmento envolvido) ou cEBRT (8 Gy, incluindo uma vértebra adicional superior e inferiormente à acometida). Pacientes com lesões epidurais mínimas a pelo menos 3 mm de distância da medula espinhal foram incluídos neste estudo. Contudo, não houve diferença significativa na resposta à dor entre SBRT e cEBRT em 3 meses (40,3% *versus* 57,9%, respectivamente), assim como nas taxas de eventos adversos. 9 A divergência entre os resultados destes dois estudos apresentados permanece alvo de discussões, reforçando a diferença biológica entre as doses utilizadas e o impacto clínico da realização de um planejamento físico com objetivos menos rigorosos.

De maneira prática, o uso da SBRT é apropriado no cenário paliativo para controle de sintomas em pacientes selecionados que apresentam metástases vertebrais dolorosas, proporcionando taxas de controle local superiores a 85 a 90% em 1 a 2 anos, mesmo em tumores radiorresistentes. Tal efeito se faz impactante para pacientes que, mesmo metastáticos, apresentam tempo de sobrevida cada vez mais prolongados conforme a evolução dos tratamentos sistêmicos. Portanto, as diretrizes recomendam o uso da SBRT principalmente para pacientes que não sejam instáveis (SINS > 12), que não apresentem doença epidural ou doença epidural mínima (Bilsky 0–1), com até 3 segmentos vertebrais contíguos no volume de tratamento e uma expectativa de vida prolongada. Por outro lado, pacientes com baixa expectativa de vida e em que o objetivo principal seja o controle da dor à curto prazo, a radioterapia convencional deve ser considerada como primeira escolha.

## Radioterapia em pacientes com oligometástases

O conceito de oligometástases foi introduzido na década de 1990 por Hellman e Weichselbaum, propondo um estado intermediário entre a doença localizada e a doença amplamente disseminada. Ele se baseia na ideia de que alguns pacientes apresentam um número limitado de metástases, que podem ser alvos de terapêuticas específicas, potencialmente alterando o curso natural da doença. Isso inclui intervenções locais, como cirurgia e radioterapia, além de terapias sistêmicas modernas. A SBRT emergiu como uma das ferramentas mais importantes no manejo das oligometástases, oferecendo alta precisão e eficácia no controle das lesões com efeitos colaterais mínimos.

Estudos randomizados de fase 2 mostraram as primeiras evidências de benefício de se realizar o tratamento direcionado para as metástases (MDT, do inglês *metastasis-directed treatment* ). Gomez et al. selecionaram apenas pacientes com até 3 metástases de neoplasia primária de pulmão não-pequenas células para receberem terapia de consolidação local (cirurgia ou SBRT) ou terapia de manutenção/observação. O estudo foi encerrado precocemente devido aos resultados favoráveis do tratamento local, com sobrevida livre de progressão (SLP) mediana de 14,2 meses ( *versus* 4,4 meses) e sobrevida global (SG) mediana de 41,2 meses ( *versus* 17 meses). 13

Em uma análise mais ampla, incluindo diferentes neoplasias primárias, o estudo SABR-COMET randomizou pacientes entre tratamento paliativo padrão isolado ou associado ao MDT com SBRT. Apesar de permitir até 5 metástases, a grande maioria (∼ 90%) apresentava até 3 lesões, sendo cerca de 33% metástases ósseas. Apesar do benefício em SLP e SG observado nos pacientes que realizaram SBRT das oligometástases, os resultados devem ser analisados de maneira individualizada devido à heterogeneidade da população estudada. 14

Diferentes fracionamentos e doses são utilizados na prática clínica para SBRT, mas as evidências sugerem que doses mais altas estão relacionadas a um maior controle local. Um estudo randomizado de fase 3 comparou os desfechos entre SBRT fracionada (27 Gy/3 frações) e de dose única (24 Gy/1 fração). Dos 117 pacientes, 88% apresentavam oligometátases ósseas e 56% localizadas em coluna vertebral. Os resultados publicados com um seguimento mediano de 52 meses mostram que a recorrência local em 3 anos foi de 5.8% com dose única e 22% com 3 frações de 9 Gy. Além disso, os pacientes tratados com dose única também apresentaram menor incidência de metástase à distância, sem apresentar aumento significativo da toxicidade. Portanto, na tentativa de melhorar o controle local deve-se considerar a prescrição de doses equivalentes altas, com análise criteriosa dos riscos de toxicidade local. 15

## Compressão medular

Com amplo espectro de sintomatologia, a compressão medular tem sua gravidade determinada de acordo com o grau de apresentação, compreendendo desde pacientes assintomáticos à plegia completa. Desta forma, é caracterizada como uma urgência oncológica e o seu manejo terapêutico é de grande importância para o controle dos danos e otimização da tentativa de reversão dos sintomas.

O uso da radioterapia neste cenário é bem consolidado e avaliado em estudos clássicos na oncologia. Em 2005, Patchell avaliou o benefício clínico do tratamento com cirurgia descompressiva e radioterapia pós-operatória. Quando comparados aos pacientes tratados exclusivamente com radioterapia, o tratamento combinado apresentou melhores desfechos quanto à capacidade neurológica funcional. No grupo de pacientes com capacidade de deambular no início do estudo, 94% no grupo cirúrgico teve preservação da função após o tratamento, em comparação a 74% no grupo de cEBRT. Dos 16 pacientes de cada braço que se encontravam incapazes de deambular, 62% recuperaram-se após o tratamento combinado, em comparação a 19% do grupo de cEBRT. Além disso, o tratamento com cirurgia e radioterapia levou à redução substancial no uso de corticosteroides e analgésicos opioides. 3

No que diz respeito à dose de radioterapia utilizada, o estudo SCORE-2 demonstrou a não-inferioridade de realizar o tratamento mais curto, com 5 frações de 4 Gy, em relação ao tratamento mais longo, com 10 frações de 3 Gy, para os pacientes com compressão epidural metastática. A redução pela metade do tempo da radioterapia não alterou o controle da dor, e também não apresentou taxa de progressão adicional ou deterioração funcional. 1 2 Na tentativa de reduzir ainda mais o tempo de tratamento, o estudo SCORAD III randomizou os pacientes entre 20 Gy em 5 frações e 8Gy em 1 fração. Apesar de não atingir o critério de não inferioridade para o estado ambulatorial em 8 semanas, que era o desfecho primário do estudo, a avaliação em 1, 4 e 12 semanas foi não inferior para a radioterapia em dose única. 16 Com isso, a depender da avaliação individualizada de cada paciente, o tratamento mais curto pode ser uma alternativa eficaz a ser considerada.

Com o intuito de melhorar o controle local, a SBRT pode ser utilizada em alguns pacientes com compressão medular. Porém, a doença epidural próxima à medula pode comprometer a probabilidade de controle e, por isso, estratégias como cirurgia de separação podem tornar o tratamento de radioterapia mais seguro e eficaz.

## Radioterapia profilática em metástases assintomáticas

Lesões ósseas metastáticas são muitas vezes diagnosticadas em exames de seguimento ou estadiamento, sem necessariamente estarem associadas a quadros álgicos. Porém, em algum momento da evolução do paciente podem ocorrer eventos ósseos relacionados a estas lesões, como fratura patológica, compressão medular ou necessidade de cirurgia ou radioterapia. O benefício da irradiação profilática em metástases ósseas foi avaliado em um estudo randomizado de fase 2 publicado recentemente, no qual cerca de 31% dos pacientes apresentavam lesões na região da coluna. A realização da cEBRT reduziu significativamente os eventos ósseos, a uma taxa de 1,6% em 1 ano, comparado a 29% nos pacientes que não foram irradiados inicialmente. Portanto, pacientes assintomáticos com lesões de alto risco, como em coluna juncional, devem ser avaliados e discutidos multidisciplinarmente quanto à melhor estratégia terapêutica. 17

## Efeitos adversos

O evento adverso mais frequente após a radioterapia de metástases ósseas dolorosas é a piora aguda do quadro álgico, com maior risco após a SBRT quando comparado à cEBRT (43% e 33%, respectivamente). 18 O aumento do risco também é observado em lesões com comprometimento de tecidos de partes moles e volume de tratamento > 8 cm ^3^ . 19

A fratura por compressão vertebral (FCV) é menos frequente, com taxas que variam entre 6 e 39% nos estudos. 20 Fatores de risco para sua ocorrência incluem lesões líticas, localização na coluna torácica, SINS > 8 pré-radioterapia, FCV preexistente, além de doses únicas elevadas (20–24 Gy). Este evento pode levar a diferentes complicações, como dor, deformidade vertebral e compressão medular, com necessidade de correção cirúrgica em alguns casos. 21

A incidência de mielopatia por radiação é baixa, atingindo valores de cerca de 0,4% em pacientes que realizaram a primeira irradiação na coluna. A dose que chega à medula é variável conforme o grau de compressão do canal vertebral, sendo maior quanto maior for a compressão. Estudos mais recentes estabeleceram limites de doses aos órgãos de risco a serem utilizados no planejamento do tratamento como tentativa de manter o risco abaixo de 5%. As plexopatias braquial e lombossacral são pouco relatadas. 22

**Fig. 1:**
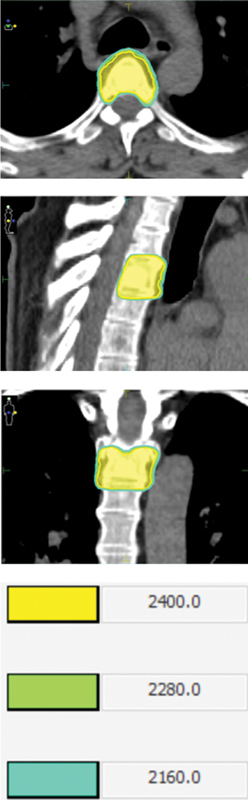
Exemplo de tratamento com radiocirurgia estereotáxica corporal (SBRT, do inglês *stereotactic body radiation therapy* ) *.* Paciente com neoplasia primária de próstata. Apresentou recidiva bioquímica, submetido a tratamento radical e em acompanhamento por 4 anos, apresentou progressão de doença em topografia óssea, com indicação de SBRT para controle local em paciente oligometastático. Foi usada a SBRT com 2 frações de 1,200 cGy na coluna torácica.

O surgimento de tratamentos que prolongam cada vez mais a sobrevida dos pacientes metastáticos aumenta a possibilidade de que, em alguns casos, seja necessária a reirradiação de lesões ósseas. Neste cenário, as toxicidades da SBRT podem se tornar mais relevantes. Da mesma forma, a associação da radioterapia com novos tratamentos sistêmicos permanece alvo de investigação, bem como de seus possíveis eventos adversos. 23 Exemplo de tratamento com SBRT ( Fig. 1 ).

## Cirurgia

Na rotina do cirurgião de coluna que trabalha no cenário oncológico, tem papel relevante o algoritmo neurológico, oncológico, mecânico, sistêmico (NOMS), desenvolvido em 2004 e atualizado a cada 5 anos com as evidências mais recentes. Este fluxograma considera os aspectos neurológico, oncológico, mecânico e sistêmico para auxiliar na melhor tomada de decisão em cada caso. 24

Em 2005, Patchell et al. 3 estabeleceram um paradigma de atuação do cirurgião de coluna no contexto da doença metastática, firmando até hoje a cirurgia como intervenção para os casos de tumores sólidos radiorresistentes que produzem compressão de alto grau do canal vertebral. O grau de compressão é avaliado nas sequências ponderadas em T2 na ressonância magnética em cortes axiais, e na escala de Bilsky pode ser compreendido como sendo: 0: apenas ósseo; 1 a, b e c: sendo compressão do saco dural sem compressão medular; 2: compressão medular ou da cauda equina mas com líquor ainda visível e 3: compressão absoluta do canal. A compressão de alto grau corresponde aos estágios 2 e 3. 25

Atualmente, as indicações cirúrgicas propostas por Patchell ainda permanecem válidas; porém, o objetivo da cirurgia para doença metastática foi atualizado com o avanço da radiocirurgia. Nos casos em que há compressão de alto grau por tumores radiorresistentes, a irradiação pode gerar mielite ou danos definitivos por proximidade anatômica e, por conta disso, faz-se necessária a abordagem cirúrgica associada em modelo de “cirurgia de separação”. Este modelo designa o procedimento no qual a ressecção do tumor é limitada à descompressão do tecido neural, criando um espaço de 2 a 3 mm, de maneira a fornecer um alvo seguro para aplicação da radioterapia local. Nessa abordagem, há ainda benefício cirúrgico por conta da menor extensão de ressecção tumoral, o que pode reduzir o tempo cirúrgico e a perda de sangue. 26

A cirurgia no contexto da doença metastática ainda considera o cenário de instabilidade mecânica. Em 2011, o SINS foi introduzido pelo SOSG para auxiliar na tomada de decisão a respeito do tratamento das lesões secundárias da coluna vertebral. De acordo com essa escala, na avaliação final, os escores podem variar de 0 a 6 denotando estabilidade, de 7 a 12 denotando instabilidade indeterminada e de 13 a 18 denotando instabilidade. Para pontuações maiores do que 12, a estabilização cirúrgica é recomendada, ao passo que, para pontuações menores do que 7, o tratamento inicial é não cirúrgico. A tomada de decisão se torna desafiadora para a maioria dos pacientes que têm pontuações entre 7 e 12 com lesões consideradas “potencialmente instáveis”. 27

## Considerações finais

Os avanços recentes no diagnóstico e no tratamento sistêmico e local do câncer metastático têm transformado a prática da oncologia, ampliando as perspectivas de sobrevida global de longo prazo para pacientes com metástases. Essa evolução traz implicações significativas para a prática da radioterapia paliativa, que passa a englobar não apenas o alívio sintomático imediato, mas também o controle prolongado da doença, com foco em minimizar os efeitos tardios da terapia e em potencialmente contribuir para a cura em pacientes selecionados.

Nesse contexto, a introdução da radioterapia estereotáxica corporal surge como uma ferramenta valiosa no manejo das metástases vertebrais, permitindo uma abordagem direcionada e eficaz. A SBRT oferece benefícios como controle duradouro da dor, prevenção da progressão local e redução de complicações associadas, além de integrar estratégias efetivas em pacientes oligometastáticos. No entanto, o uso de doses escalonadas deve ser cuidadosamente ponderado, considerando-se o potencial risco de toxicidade aumentada, a necessidade de tecnologia avançada e equipe treinada, e o tempo necessário para a aplicação, em comparação à radioterapia convencional.

Portanto, a SBRT representa um avanço promissor no manejo das metástases da coluna, especialmente em pacientes selecionados, alinhando-se às demandas de uma oncologia moderna que busca conciliar eficácia terapêutica, preservação da qualidade de vida e abordagens individualizadas.
